# House Flies (*Musca domestica*) from Swine and Poultry Farms Carrying Antimicrobial Resistant Enterobacteriaceae and *Salmonella*

**DOI:** 10.3390/vetsci10020118

**Published:** 2023-02-04

**Authors:** Fabrizio Bertelloni, Flavio Bresciani, Giulia Cagnoli, Bruno Scotti, Luca Lazzerini, Marco Marcucci, Giuseppe Colombani, Stefano Bilei, Teresa Bossù, Maria Laura De Marchis, Valentina Virginia Ebani

**Affiliations:** 1 Department of Veterinary Science, University of Pisa, Viale delle Piagge 2, 56124 Pisa, PI, Italy; 2 Azienda Usl Toscana Nord Ovest, Sede Sicurezza Alimentare e Sanità Pubblica Veterinaria, Zona Versilia, Via Martiri di S. Anna 12, 55045 Pietrasanta, LU, Italy; 3 Azienda Usl Toscana Nord Ovest, Sede Sicurezza Alimentare e Sanità Pubblica Veterinaria, Zona Valle del Serchio, Via IV Novembre 10, 55027 Gallicano, LU, Italy; 4 Istituto Zooprofilattico Sperimentale del Lazio e della Toscana M. Aleandri, 00178 Rome, RM, Italy

**Keywords:** house fly (*Musca domestica*), Enterobacteriaceae, *Salmonella* spp., antimicrobial resistance

## Abstract

**Simple Summary:**

House flies (*Musca domestica*) are ubiquitous insects living in close contact with humans and farmed animals. Due to their behavior and life cycle, these insects could be easily contaminated by bacteria, becoming mechanical vectors of potentially pathogenic microorganisms. In the present investigation, house flies were captured in poultry and swine farms, and they were microbiologically evaluated. Enterobacteriaceae were abundantly detected in analyzed samples; high levels of resistance were found against commonly used antimicrobials, such as β-lactams and tetracyclines. One extended spectrum β-lactamase producer strain was identified among Enterobacteriaceae, carrying the gene blaTEM-1. *Salmonella* spp. was detected in samples from about one third of farms; most of the tested antimicrobials were effective against more than 80% of isolated salmonellae. House flies could be important vectors of antimicrobial resistant bacteria and *Salmonella*, representing a potential source of infection for farmed animals and practitioners.

**Abstract:**

The house fly (*Musca domestica*) is a very common insect, abundantly present in farm settings. These insects are attracted by organic substrates and can easily be contaminated by several pathogenic and nonpathogenic bacteria. The aim of this survey was to evaluate the presence of *Salmonella* spp. and other Enterobacteriaceae in house flies captured in small-medium size farms, located in Northwest Tuscany, Central Italy, and to evaluate their antimicrobial resistance; furthermore, isolates were tested for extended spectrum β-lactamase and carbapenems resistance, considering the importance these antimicrobials have in human therapy. A total of 35 traps were placed in seven poultry and 15 swine farms; three different kinds of samples were analyzed from each trap, representing attractant substrate, insect body surface, and insect whole bodies. Enterobacteriaceae were isolated from 86.36% of farms, 82.87% of traps, and 60.95% of samples; high levels of resistance were detected for ampicillin (61.25% of resistant isolates) and tetracycline (42.5% of resistant isolates). One extended spectrum β-lactamase producer strain was isolated, carrying the *bla*_TEM-1_ gene. *Salmonella* spp. was detected in 36.36% of farms, 25.71% of traps, and 15.24% of samples. Five different serovars were identified: Kentucky, Kisarawe, London, Napoli, and Rubislaw; some isolates were in R phase. Resistance was detected mainly for ampicillin (31.21%) and tetracycline (31.21%). House flies could represent a serious hazard for biosecurity plans at the farm level, carrying and sharing relevant pathogenic and antimicrobial resistant bacteria.

## 1. Introduction

Antimicrobial resistance (AMR) is a serious threat to public health. This problem arose with particular strength in the past decades, and some authors supposed that, without corrective interventions, we could come back to a pre-antibiotic era [1,2]. Massive use of antimicrobials in food-producing animals could strongly contribute to this issue and, in a One Health view, specific programs and plans were adopted and implemented worldwide to reduce the use of antimicrobials in livestock [3].

It was demonstrated that a good biosecurity plan can decrease antimicrobial use [4,5] and, consequently, the spreading of antimicrobial resistant bacteria [6]. Biosecurity in animal production systems represents a set of practices to manage the risks of infectious disease introduction and spreading within and between farms [7,8]. These “good practices” include many different actions that farmers could adopt to fight infectious diseases [7].

One of the actions required for a good biosecurity plan is the control of pest animals that could contribute to the introduction and/or the spread of several pathogens within the farm. Indeed, rodents, insects, and wild birds can act as reservoirs and asymptomatic carriers of many pathogens [9,10,11,12], and they can be the source of disease agents for farmed animals [13,14].

Among pests, house flies (*Musca domestica*) represent an important threat to biosecurity plans. House flies are largely dispersed in every habitat; they are attracted by biological materials, in particular, waste and feces, and they are a common inhabitant of every farm environment. As a consequence, their eradication is impossible and a reduction to acceptable levels is the only feasible objective at the farm level [15]. Although different control strategies are developed, such as traps or insecticides, none has a full efficacy, and an integrated approach is necessary [16,17]. Houseflies can acquire and share different enteric bacterial pathogens, such as *Salmonella* and *Campylobacter* [10,18]. In particular, houseflies can easily acquire *Salmonella* from food, feed, manure, and waste; furthermore, adult insects can transmit this bacterium to each other, and vertical transmission is demonstrated too [18,19]. Additionally, houseflies may move antimicrobial resistant bacteria [20]. In particular, it has been suggested that these insects contribute to the spread of and maintain antimicrobial resistant bacteria within a herd [21,22]. Most of the studies focused on Gram negative bacteria, in particular, *Escherichia coli* and other Enterobacteriaceae [21,22,23,24], but some surveys involving Gram positive bacteria, such as *Staphylococcus* spp., have been performed too [25].

The aim of this study was: (1) to investigate the contamination by *Salmonella* spp. and other Enterobacteriaceae in house flies collected in medium-size poultry and swine farms; (2) to assess antimicrobial resistance in the bacterial isolates.

## 2. Materials and Methods

### 2.1. Farms

Poultry and swine monospecies farms were selected for this study. All farms were located in Northwest Tuscany, Central Italy, under the area of competence of “Az. USL di Versilia, Valle del Serchio e Piana di Lucca”. All farms were small/medium farms with family management. Poultry farms were intensive, indoor, free-range breeding of laying hens; about 2000–3000 animals were present. Swine farms were semi-extensive, open-cycle, indoor breeding of about 10–100 animals.

Sampling operations were carried out during the summer of 2019, from late June to early September. Each farm was sampled once. 

### 2.2. Traps, Insects Collection, and Processing

Homemade traps were used to catch the flies. Traps were prepared in the Laboratory of Infectious Disease, Department of Veterinary Science, University of Pisa. Empty glass jars with airtight caps were sterilized in autoclave. Fish broth was employed as an attractant; in particular, commercial fish for human consumption were boiled in potable water for 3 h. The obtained broth was filtered to remove gross material and boiled again in a sterile bottle. The final broth was checked for sterility by culturing 1 mL on Brain Heart Infusion broth (BHI) (Thermo Fisher Diagnostics, Milan, Italy) for 24 h at 37 °C; it was stored at 4 °C, for a maximum of 3 days, until it was used. Traps were assembled in the laboratory, in sterile condition, during the same day when they would be placed in the selected farms. Each sterilized glass jar was filled with 50 mL of sterile broth.

Traps, transported to farms in refrigerated conditions, were located inside the animal breeding rooms, far from windows and doors, and not accessible to the animals. One trap was located in each breeding room.

After about 24 h, traps were removed, closed, and transported to the Laboratory of Infectious Disease, in refrigerated conditions, where they were processed within 4 h. From each trap, 3 different samples (A, B, C) were obtained.

Sample A: 4 mL of broth was collected from the jar and stored in a sterile tube.

Sample B: all insects were removed with sterile instruments and placed in a sterile petri dish; house flies (*M. domestica*) were morphologically identified [26], and 10 from each trap were randomly selected and transferred into a tube containing 5 mL of sterile saline water. Tubes were vigorously vortexed for 30 s; 4 mL of the solution (sample B) were transferred into a new tube.

Sample C: insect bodies from the previous step were transferred, with new sterile instruments, into a tube containing 20 mL of 70% ethanol; after 5 min of gentle shaking, insects were placed in a sterile petri dish and left to dry in a biosafety cabinet. Subsequently, fly’s bodies were homogenized in 5 mL of sterile saline water with stomacher, and 4 mL of homogenate (sample C) were transferred into a new tube.

Sample A reflects the contamination due to the insects, sample B represents the investigated bacteria colonizing the surface body of the captured house flies, and sample C provides data about the investigated bacteria present inside the flies.

### 2.3. Enterobacteriaceae Isolation

In order to isolate bacteria belonging to the Enterobacteriaceae family, 1 mL of each sample was diluted from 10^−1^ to 10^−3^ in sterile saline water. Successively, 0.1 mL from each dilution and from the original sample were inoculated with the spread-plate method on Violet Red Bile Glucose Agar (VRBGA) (Thermo Fisher Diagnostics, Milan, Italy) in order to obtain single isolated colonies; plates were incubated at 37 °C for 24 h. Up to 3 distinct and apparently different colonies from the VRBGA of each sample were selected and purified on Tryptic Soy Agar (TSA) (Thermo Fisher Diagnostics, Milan, Italy). Isolates were confirmed by Gram staining, oxidase, and catalase tests, and they were additionally tested for lactose fermentation with Violet Red Bile Agar (VRBA) (Thermo Fisher Diagnostics, Milan, Italy) and glucuronidase, with Tryptone Bile X-Gluc (TBX) AGAR (Biolife, Milan, Italy). One to 3 different isolates from each sample were selected on the basis of lactose fermentation and glucuronidase activity. All selected isolates were cultured in BHI and frozen at −80 °C by the addition of 20% glycerol.

### 2.4. Salmonella Isolation and Identification

*Salmonella* spp. isolation was carried out, as previously described, with few modifications [27]. Briefly, 1 mL from each kind of sample (A, B, C) was inoculated into 9 mL of Buffered Peptone Water (BPW) (Thermo Fisher Diagnostics, Milan, Italy) and incubated at 37 °C for 24 h. From BPW, 1 ml and 0.1 mL were subcultured in 10 mL of Selenite broth (Biolife, Milan, Italy) and 10 mL of Rappaport-Vassiliadis (RV) broth (Biolife, Milan, Italy), respectively. Selenite broth was incubated at 37 °C, whereas RV broth was incubated at 41.5 °C. After 24 h of incubation, a loopful from each broth was streaked on Brilliant Green Agar (Biolife) and Salmonella-Shigella Agar (Biolife) plates; plates were incubated at 37 °C for 24 h. Suspected colonies were evaluated with conventional biochemical tests (Triple Sugar Iron Agar, urease, ONPG, lysin decarboxylase, indole, VP, malonate) and were confirmed as *Salmonella* spp. by detection of *invA* gene [28]. Isolates belonging to the *Salmonella* genus were serotyped according to the Kaufmann–White *Salmonella* serotyping scheme with commercial antisera (Statens Serum Institut, Copenhagen, Denmark). All identified strains were cultured in BHI and frozen at −80 °C by the addition of 20% glycerol.

### 2.5. Antimicrobial Susceptibility Tests

Obtained isolates were tested for antimicrobial resistance with the disk diffusion method, as described by CLSI [29]. Briefly, a suspension equivalent to 0.5 McFarland turbidity was prepared in sterile saline water for each isolate, and it was inoculated onto Mueller–Hinton agar (MH) (Thermo Fisher Diagnostics, Milan, Italy) plates with a sterile cotton swab. A maxim of 6 antimicrobial disks were placed in each plate and, subsequently, MH plates were incubated at 35 °C for 16–20 h. The antimicrobials disks (Thermo Fisher Diagnostics, Milan, Italy) tested were as follows: ampicillin (10 µg), amoxicillin-clavulanate (20/10 μg), cefoxitin (30 μg), cefotaxime (30 μg), ceftiofur (30 μg), Imipenem (10 μg), ertapenem (10 μg), aztreonam (30 μg), chloramphenicol (30 μg), tetracycline (30 μg), enrofloxacin (5 μg), ciprofloxacin (5 μg), gentamicin (10 μg), amikacin (30 μg), and trimethoprim-sulfamethoxazole (1.25/23.75 μg). Results were interpreted in accordance to CLSI guidelines [30,31].

Enterobacteriaceae isolates, including *Salmonella*, which were resistant or intermediate to Imipenem or ertapenem in the disk diffusion test, were evaluated for Carbapenemase production with the Modified Carbapenem Inactivation Methods (mCIM) and EDTA-modified Carbapenem Inactivation Methods (eCIM) [31]. The same isolates were tested for *bla*_NDM_, *bla*_KPC_, *bla*_OXA-48_, *bla*_IMP_, and *bla*_VIM_ gene presence too. Primers and PCR protocols, previously described by other authors, were adopted [32].

Enterobacteriaceae, including *Salmonella*, showing, in the disk diffusion test, an inhibition zone diameter lower than 27 mm for cefotaxime and/or aztreonam, were tested for ESBL production, as reported by CLSI [31]. Furthermore, the occurrence of genes *bla*_TEM_, *bla*_SHV_, and *bla*_CTX-M_ were evaluated with primers and PCR protocols, as described in the literature [33,34]. 

Enterobacteriaceae, including *Salmonella*, which were resistant or intermediate to cefoxitin, were tested for AmpC β-lactamases using a combination disk test with cefotaxime (30 μg), ceftazidime(30 μg), and cloxacillin (500 μg) [35]. These isolates were further tested by PCR for *bla*_CMY-1_ and *bla*_CMY-2_ gene presence, as previously reported [34]. 

Positive PCR products were sequenced (BMR Genomics, Padova, Italy); obtained sequences were analyzed using BioEdit and compared with gene banks database using Basic Local Alignment Search Tool (BLAST) and FASTA (https://www.ebi.ac.uk/Tools/sss/fasta/) (accession date 7 October 2022).

### 2.6. Statistical Analyses

The Chi-square (X^2^) test was employed to compare the obtained results. In particular, we evaluated the differences in the isolation rates of Enterobacteriaceae and *Salmonella* between poultry and swine farms, and among the different types of samples (A, B, C). Furthermore, the level of antimicrobial resistance detected was compared between isolates cultured from poultry and swine farms, and among isolates coming from the different types of samples (A, B, C). The statistical significance threshold was set at *p*-value ≤ 0.05.

## 3. Results

### 3.1. Farms and Samples

Twenty-two farms were enrolled in the study: seven and 15 poultry and swine farms, respectively. Thirty-five traps were placed: 10 in poultry and 25 in swine farms (Table 1). All the traps allowed the capture of insects. Furthermore, only house flies were found in all traps; the number of *M. domestica* specimens recovered in the traps ranged between eight (Trap ID 8) and 30. Overall, 105 samples were processed for microbiological investigation: 35 samples each for type A, B, and C.

### 3.2. Enterobacteriaceae

Enterobacteriaceae were successfully isolated from 29/35 (82.87%) traps collected in 19/22 (86.36%) farms (Table 1). Enterobacteriaceae were obtained in pure culture from 64/105 (60.95%) samples: 20/35 (57.14%) type A, 20/35 (57.14%) type B, and 24/35 (68.57%) type C samples. In 9/105 (8.57%) samples (6 type A and 3 type B), bacterial overgrowth did not allow for the selection of single isolated colonies; these samples were not processed further, nor were they included in the study. No statistical differences emerged among the positivity rate of the three kinds of samples (*p* = 0.52), considering the “overgrowth samples” too. It was possible to isolate Enterobacteriaceae from all three samples (A, B, C) coming from 12/29 (41.38%) of the positive traps (12/35, 34.29%, of the total); considering the “overgrowth samples” as positive, the total number of positive traps, where it was possible to detect Enterobacteriaceae in all samples, was 17/29 (58.62%) (17/35, 48.57%, of the total). Enterobacteriaceae were isolated in 9/10 (90.00%) farms where more than one trap was placed; it was possible to detect Enterobacteriaceae in all traps in 7/10 (70.00%) farms. Ten out of twelve (83.33%) farms, where only one trap was placed, scored positive. No statistical difference emerged between the positivity rate of farms where one trap was placed and farms where more than one trap was placed (*p* = 0.65).

Eighty different isolates belonging to Enterobacteriaceae were obtained from the 64/105 positive samples and submitted to antimicrobial tests. Particularly, 24, 25, and 31 isolates were obtained from type A, B, and C samples, respectively.

Considering animal sites where flies were captured, 12/15 (80.00%) farms, 19/25 (76.00%) traps, and 40/75 (53.33%) samples from swine settings were positive. Furthermore, 7/7 (100%) farms, 10/10 (100%) traps, and 24/30 (80.00%) samples from poultry locations were positive. No differences emerged considering the number of farms and traps that scored positive (*p* = 0.20 and *p* = 0.08, respectively), whereas samples from poultry were found to be positive more often than the ones from swine (*p* = 0.01).

Results of the disk diffusion antimicrobial test are shown in Table 2. High levels of resistance were detected for ampicillin (61.25%) and tetracycline (42.5%). The most effective antimicrobials were ertapenem (96.25% of susceptible isolates), trimethoprim-sulfamethoxazole (91.25% susceptible isolates), aztreonam (88.75% of susceptible isolates), ciprofloxacin (85.00% of susceptible isolates), and gentamicin (88.75% of susceptible isolates). A higher percentage of ceftiofur resistant Enterobacteriaceae were detected among isolates from hens, rather than among those from swine (*p* = 0.01).

Forty-four isolates were tested for carbapenemase production; none showed positive results. Four isolates were classified as intermediate, presenting an inhibition zone between 16 and 18 mm or the presence of pinpoint colonies within an inhibition zone ≥19 mm. All 44 isolates, screened for resistance genes (*bla*_NDM_, *bla*_KPC_, *bla*_OXA-48_, *bla*_IMP_, and *bla*_VIM_) were negative. All tested isolates were classified as non carbapenemase producers.

Sixty-six isolates were evaluated for ESBL production and ESBL gene (*bla*_TEM_, *bla*_SHV_, *bla*_CTX-M_) presence. Six isolates gave intermediate results with a phenotypic test, showing an increase ≥ 5 mm in the inhibition zone diameter for only one antimicrobial agent (cefotaxime or ceftazidime), tested in combination with clavulanate vs the zone diameter of the agent tested alone. Only one isolate (*E. coli*) tested positive for the gene *bla*_TEM_, belonging to *bla*_TEM-1_; it was classified as an ESBL producer.

Nineteen and nine isolates, resistant and intermediate to cefoxitin, respectively, were tested for AmpC β-lactamases production. All isolates were negative in the phenotypic test. The genes *bla*_CMY-1_ and *bla*_CMY-2_ were not detected; thus, none of the isolates were considered AmpC β-lactamase producers.

Enterobacteriaceae isolates were resistant from zero to seven different antimicrobials. In particular, 26/80 (32.5%) were classified as multi-drug resistant (MDR), due to being resistant to one or more antimicrobials in at least three different antimicrobial classes [36]. Multi-drug resistance was detected in 9/32 (28.12%) and 17/48 (35.42%) poultry and swine isolates, respectively; no statistical differences emerged (*p* = 0.49). Regarding the kind of sample, 8/24 (33.33%), 8/25 (32.00%), and 10/31 (32.26%) MDR isolates were detected in A, B, and C samples, respectively; no statistical differences emerged (*p* = 0.99). Table 3 shows the relevant information on MDR isolates and their antimicrobial resistance profile.

### 3.3. Salmonella *spp*.

*Salmonella* spp. were detected in 8/22 (36.36%) farms; all, but one, were swine farms (Table 1). No statistical differences emerged between poultry and swine farm positivity (*p* = 0.14). *Salmonella* was isolated from 16/105 (15.24%) samples, derived from 9/35 (25.71%) traps. *Salmonella* was isolated from more than one trap, placed in the same farm, in only one case (Farm 21). Five, six, and three samples of the types A, B, and C were positive, respectively; no statistical differences emerged (*p* = 0.56). From two samples (one type A and one type C), two different *Salmonella* serovars were isolated. *Salmonella* was generally found in only one kind of sample (A, B, or C) from each trap. It was isolated from all three types of samples in only two cases: Traps ID 13 and 28. However, the serovars detected in the three samples were different. In one case (Trap ID 12), *Salmonella* was detected in sample A and B. Five different *Salmonella* serovars were detected: Kentucky, Kisarawe, London, Napoli, and Rubislaw. Furthermore, three isolates were in R phase, and they cannot be serotyped. Table 4 shows detailed information about *Salmonella* spp. positive samples.

Table 5 reports data about the antimicrobial resistance of *Salmonella* isolates. A low susceptibility was recorded only for ampicillin, ceftiofur, and tetracycline. More than 70% of isolates were susceptible to the other antimicrobials tested. Three *Salmonella* isolates, intermediate to Imipenem and one *Salmonella* isolate resistant to Imipenem, were tested for carbapenemase production, and all were negative; the same isolates, tested with PCR, were negative for carbapenem resistance genes. Eleven *Salmonella* isolates were negative for the ESBL production and the presence of *bla*_TEM_, *bla*_SHV_, and *bla*_CTX-M_ genes. 

*Salmonella* isolates were resistant from zero to seven different antimicrobials; three isolates were classified as MRD (Table 4). 

## 4. Discussion

House flies are very common and widespread insects that are largely present in farm environments [16,37]. Although *M. domestica* is not an ectoparasite, and it does not feed on animals, it could directly represent an irritation cause for farmed animals [16]. Furthermore, this insect feeds on feces, manure, and waste; thus, it may be important vectors of different pathogens and antimicrobial resistant bacteria [20,37].

The potential role of house flies in the spreading of *Salmonella* and antimicrobial resistant Enterobacteriaceae, in medium-size swine and poultry farms, has been investigated in the present study.

First, a trap was developed to allow the capture of house flies. Different synthetic or natural attractive substrates were tested (data not shown), but a homemade fish broth gave the best results and allowed for the capture of *M. domestica* insects only.

Different kinds of samples were evaluated. Sample A, the broth present in the trap, provided information about the contamination carried out by insects. Sample B represented the Enterobacteriaceae colonizing the surface body of the captured house flies. Finally, sample C provided data about the internal Enterobacteriaceae of the flies. Obtained data showed that all these samples should be evaluated to have a full picture of the bacteria shared by house flies in farm environments. Indeed, in several cases, it was possible to isolate bacteria only from one or two kinds of samples. A possible issue with the proposed trapping method could be the cross-contamination between samples A and B. However, considering the acquired results, this event seems to not be frequent. Particularly, data about *Salmonella* spp. isolation showed that there was not correspondence in the isolation rate among the three kinds of samples: positivity of sample A was not always associated to positivity of samples B, and vice versa. Furthermore, in some cases, different serovars were isolated from the samples coming from the same trap.

Enterobacteriaceae are ubiquitous bacteria, common inhabitants of the intestines of animals; they can successfully survive in the environment and can be considered an index of fecal contamination [38,39].

In the present investigation, Enterobacteriaceae were isolated from more than 80% of traps and from almost all farms (19/22). Furthermore, Enterobacteriaceae were often isolated from all three kinds of samples. These data confirm the wide distribution of these bacteria. No differences were found in positivity rates among swine and poultry farms and traps; however, samples from hens were more often positive than samples from swine. Our data confirm the data present in the available literature, showing that house flies could easily become contaminated by Enterobacteriaceae, probably by contact with feces or animal bodies. Although many studies only focused on *E. coli*, several authors reported a high isolation rate of Enterobacteriaceae from *Musca domestica*, frequently close to 100% [21,24,40].

Most of the isolates were resistant to ampicillin (61.25%) and tetracycline (42.50%). In Italy, as well as in Europe, penicillins and tetracyclines were the most sold compounds in 2020 for producing animals, accounting for about 50–60% of the total [41]. The detected resistance rate could be the consequence of the abundant use of these antimicrobials in livestock. Other authors reported that Enterobacteriaceae isolated from house flies were frequently resistant to ampicillin and tetracyclines; in detail, some studies described higher percentages of resistant isolates [24,40,42] and others found lower [25] or very similar percentages [21] of resistant isolates in comparison with our results. 

Ertapenem (96.25% of susceptible isolates), trimethoprim-sulfamethoxazole (91.25% of susceptible isolates), aztreonam (88.75% of susceptible isolates), ciprofloxacin (85.00% of susceptible isolates), and gentamicin (88.75% of susceptible isolates) were the most effective antimicrobials against the tested Enterobacteriaceae isolates. This is in line with the results reported by other authors [25,42,43], with few works reporting higher percentages of resistant isolates [24].

No differences were detected regarding antimicrobial resistance between isolates from poultry and swine farms; however, ceftiofur resistance was more often detected in isolates from hens. We can hypothesize a more frequent and abundant use of ceftiofur in this animal species. 

Extended spectrum beta-lactamases, carbapenemases, and AmpC type b-lactamases are enzymes produced by bacteria that are able to hydrolyze many β-lactam antimicrobials, and they are mainly produced by Gram-negative bacteria, particularly members of the Enterobacteriaceae family [44,45]. Β-lactams are antimicrobials commonly used in human medicine to treat different kinds of infections, and some of them, such as extended spectrum cephalosporin and carbapenems, are considered last-resort antimicrobials for the treatment of highly drug-resistant bacteria [46,47]. Consequently, extended-spectrum β-lactamase, carbapenemase, and AmpC-producing Enterobacteriaceae represent a serious threat to human medicine due to the difficulty to arrange an effective antibiotic therapy in case of infection [48]. 

In the present investigation, no carbapenemases and AmpC β-lactamase producers were detected. Few studies explored this aspect in bacteria from house flies collected in producing animal settings. Poudel and collaborators reported one *bla_CMY-2_* positive *E. coli* out of 84 tested isolates from house flies [25]. In the survey by Alves and colleagues, a low detection of AmpC β-lactamase producing strains was described [21]. Bakry and coworkers showed the wide presence of *bla_CMY-2_* positive *E. coli* (61.5%) in *M. domestica* collected in cattle environment; the same authors did not find *bla_OXA_* positive isolates [24]. 

Only one ESBL producer isolate, carrying *bla_TEM-1_*, was detected. This gene, conferring resistance to ampicillin, penicillin, and first-generation cephalosporins, was initially detected in 1965; many *bla_TEM_* variants exist and they are largely dispersed worldwide, even if, in recent years, different kinds of ESBL genes increased [45,48]. In other investigations on Enterobacteriaceae from flies, a higher percentage of ESBL strains carrying *bla_TEM_* was reported, and the presence of other genes was described too. A study found 76.9% of tested isolates positive for the gene *bla_TEM_*; furthermore, 53.8% and 19.2% had *bla_SHV_* and *bla_CTX-M_*, respectively [24]. Alves and collaborators reported percentages of 36.3% and 11.1% of *bla_TEM_* and *bla_CTX-M_* positive isolates, respectively [21]. Onwugamba and coworkers investigated a subset of 16 phenotypically-detected ESBL Enterobacteriaceae, cultured from flies; all isolates harbored *bla_CTX-M_* and 33% of them had *bla_TEM_* too. These authors reported *bla_TEM-1_* as the predominant variant [49]. On the other hand, Poudel and collaborators showed results similar to the ones obtained in our study; indeed, among 84 tested *E. coli* and 37 *Klebsiella pneumoniae* tested, three and one were phenotypically ESBL positive, respectively. *K. pneumoniae* carried the *bla_CTX-M-1_* gene, whereas *E. coli* were positive for *bla_TEM_*; *bla_TEM-1_* was the detected variant in this case too [25].

The concurrent resistance to multiple antimicrobials offers a great advantage to bacteria. In our study, most of the tested isolates were resistant to only a few different antimicrobials and antimicrobial classes; however, 32.50% were classified as MDR, with no differences between isolates obtained from flies collected in poultry and swine farms, and among isolates coming from the three kinds of samples. The percentages of MDR strains found in flies by other authors are variable in relation to the geographic area and sites of insect collection; however, with few exceptions, the available data are in line with our results. Bakry and collaborators detected a percentage of 100% of MDR *E. coli* isolated from *M. domestica* specimens collected in a dairy farm [24]. In another survey on MDR isolates carried by flies in dairy farms, the percentages of MDR *E. coli* ranged between 3.17% and 44.44% [21]. In a study on antimicrobial resistance in bacteria isolated from house flies, collected in different settings, the percentage of MDR Enterobacteriaceae ranged between 5.9% and 50% [43]. Poudel and coworkers identified 19.04% of MDR *E. coli* and 5.4% of MDR *K. pneumoniae* among isolates coming from flies collected in different locations [25]. 

The presence of *Salmonella* was evaluated in our study as well. Salmonellosis is the second zoonosis in Europe in terms of the number of human cases and, in 2019, ‘eggs and egg products’ and ‘pig meat and products thereof’ were the first and third source of human infections, responsible for 37.0% and 9.8% of the total strong-evidence outbreaks, respectively [50].

As expected, *Salmonella* was isolated less frequently than Enterobacteriaceae, with only 15.24% of positive samples. However, it was isolated from 25.71% of traps, coming from 36.36% of farms; these results stress the importance of analyzing the different kinds of samples to detect this pathogen. Furthermore, obtained data confirmed the spreading of *Salmonella*, mainly in swine farms.

As reported by other authors, house flies can easily be colonized by *Salmonella* [18] and these insects could be responsible for the dispersion of this bacterium, particularly within a farm. 

Five different *Salmonella* serovars were detected in our study (Kentucky, Kisarawe, London, Napoli, Rubislaw). None of the six most frequently reported serovars involved in human salmonellosis in 2019 were detected; they are not the most commonly found serovars in swine in the EU [50], as well as in Italy [51,52]. This could reflect the different epidemiological scenarios of the investigated farms that, as reported, were medium-size farms, with a reduced number of animals. On the other hand, the identification of *S.* ser. Kentucky in the poultry farm is in line with the national prevalence of this serotype in the years 2016–2018 [53], and its finding in other poultry farms of the Tuscany region in 2019 (personal data of the Regional Reference Center—Centro di Riferimento regionale per gli Enterobatteri Patogeni).

Resistance to antimicrobials is less dispersed in *Salmonella* than in other bacteria. Indeed, in 2019 in Europe, only 25% of human isolated salmonellae were multi-drug resistant, and most isolates were susceptible to all tested antimicrobials; in isolates from food producing animals, the proportion of resistant strains is higher than in humans ones, but there remains a high number of *Salmonella* isolates susceptible to all tested molecules [54]. Furthermore, a high level of resistance is generally detected only in more virulent serovars, such as typhimurium, typhimurium monophasic variant, and enteritidis [52,54]. Data obtained in the present study seem to confirm this statement; most of the tested antimicrobials were effective against more than 80% of isolates. A reduced susceptibility was recorded only for ampicillin, ceftiofur, and tetracycline. These data are in line with the recent EFSA report [54], and other investigations carried out in central Italy, on salmonellae from swine [51,52]. These antimicrobials are widely employed in livestock, and this could be the cause of these findings. No ESBL and carbapenemase producers salmonellae were detected in our investigation, accordingly with EU data [54] and recent studies [55,56].

## 5. Conclusions

House flies are common pests associated with poor hygienic conditions. Furthermore, these insects can serve as mechanical vectors of several pathogens. 

The present research confirmed that house flies living in medium-small swine and poultry farms are frequently contaminated with Enterobacteriaceae. Our data highlighted the importance of *M. domestica* as a potential spreader of antimicrobial resistant bacteria in small farms as well. High levels of resistance were detected mainly for those antimicrobials frequently employed to treat livestock infections, probably as a consequence of selection pressure at the farm level. Moreover, commensal and opportunistic Enterobacteriaceae carried by flies could serve as reservoirs and a source of resistance genes for pathogenic bacteria too. Furthermore, the obtained results highlight the importance that house flies could have in the spreading of ESBL resistant bacteria, representing a serious problem for public health. 

House flies were contaminated by *Salmonella* spp. too. These insects can be responsible for the spreading and maintenance of this pathogen in a farm environment, representing a source of infection for animals and humans.

Microbiological controls on captured house flies could give useful information about pathogens and antimicrobial resistant bacteria circulating in farms, and about the level of antimicrobial resistances. The proposed method, with different kinds of samples to be analyzed, will give a more complete picture. The attractant substrate that remains in the traps reflects the environmental contamination. The positivity of the fly’s surface body shows that *M. domestica* can serve as a mechanical vector of antimicrobial resistant and pathogenic bacteria. The insect body positivity suggests that house flies could be “biological vectors” too, carrying and excreting these bacteria for long periods and eventually transmitting them vertically.

## Figures and Tables

**Table 1:** Positive farms and traps to Enterobacteriaceae and *Salmonella*.

Farm	Farmed Animals	Traps ID	Number of Positive Traps to Enterobacteriaceae	Positive Samples to Enterobacteriaceae			Number of Positive Traps to *Salmonella*
				A	B	C	
1	Poultry	1	2/2	+	+	+	0/2
		2		+	-	-	
2	Swine	3	1/1	+	+	+	0/1
3	Swine	4	1/1	+	+	+	0/1
4	Poultry	5	1/1	+	+	+	0/1
5	Poultry	6	2/2	+	+	+	0/2
		7		*	+	+	
6	Poultry	8	1/1	+	+	+	0/1
7	Poultry	9	1/1	+	+	+	0/1
8	Swine	10	2/2	+	+	+	1/2
		11		-	-	+	
9	Swine	12	0/1	*	-	-	1/1
10	Swine	13	1/1	-	+	+	1/1
11	Poultry	14	2/2	+	+	-	1/2
		15		*	*	+	
12	Swine	16	1/1	+	+	+	0/1
13	Swine	17	1/2	-	-	-	0/2
		18		+	-	+	
14	Swine	19	1/1	-	+	+	0/1
15	Swine	20	0/1	-	-	-	0/1
16	Swine	21	1/1	+	+	-	0/1
17	Poultry	22	1/1	+	+	+	0/1
18	Swine	23	3/3	+	+	+	0/3
		24		+	+	+	
		25		+	-	+	
19	Swine	26	2/2	*	*	-	1/2
		27		-	+	+	
20	Swine	28	1/2	*	*	-	1/2
		29		-	-	-	
21	Swine	30	3/3	*	+	+	2/3
		31		+	+	+	
		32		+	-	-	
22	Swine	33	3/3	+	-	-	1/3
		34		-	-	+	
		35		-	-	+	

Legend. ID: identification number; * samples where the overgrowth of bacteria did not allow us to obtain pure culture isolates.

**Table 2:** Results of disk diffusion test for Enterobacteriaceae.

Antimicrobial	Susceptible		Intermediate		Resistant	
	N° of Isolates	%	N° of Isolates	%	N° of Isolates	%
Ampicillin	17	21.25	14	17.50	49	61.25
Amoxicillin-clavulanate	42	52.50	17	21.25	21	26.25
Cefoxitin	52	65.00	9	11.25	19	23.75
Cefotaxime	21	26.25	41	51.25	18	22.50
Ceftiofur	39	48.75	32	40.00	9	11.25
Imipenem	37	46.25	27	33.75	16	20.00
Ertapenem	77	96.25	3	3.75	0	0.00
Aztreonam	71	88.75	7	8.75	2	2.50
Chloramphenicol	54	67.50	20	25.00	6	7.50
Tetracycline	44	55.00	2	2.50	34	42.5
Enrofloxacin	55	68.75	19	23.75	6	7.50
Ciprofloxacin	68	85.00	12	15.00	0	0.00
Gentamicin	68	85.00	6	7.50	6	7.50
Amikacin	47	58.75	30	37.50	3	3.75
Trimethoprim-sulfamethoxazole	73	91.25	1	1.25	6	7.50

**Table 3:** Multi-drug resistant Enterobacteriaceae isolates.

Farm	Farmed Animals	Traps ID	Samples Type	Resistance Profile
1	Poultry	1	B	AMP IMP C TE
1	Poultry	2	A	AMP TE ENR
1	Poultry	2	A	AMP FOX CTX TE ENR
2	Swine	3	A	AMP IMP TE SXT
2	Swine	3	B	AMP AMC ATM TE
3	Swine	4	C	FOX CTX EFT ATM TE
4	Poultry	5	A	AMP CTX EFT TE AK
4	Poultry	5	B	AMP IMP TE
5	Poultry s	6	C	AMP CTX EFT IMP TE ENR
6	Poultry	8	C	AMP AMC FOX IMP
7	Poultry	9	C	AMP FOX EFT ENR
8	Swine	11	C	AMP AMC FOX CTX TE
10	Swine	13	C	AMP AMC CTX IMP C TE SXT
12	Swine	16	B	IMP TE SXT
12	Swine	16	C	IMP TE SXT
13	Swine	18	A	AMP C TE SXT
13	Swine	18	C	CTX IMP TE
16	Swine	21	A	AMP AMC FOX IMP
16	Swine	21	B	AMP AMC IMP TE ENR
17	Poultry	22	A	AMP IMP C TE SXT
18	Swine	25	A	AMP AMC C TE
19	Swine	27	B	AMP CTX CN
19	Swine	27	C	AMP AMC FOX TE CN
21	Swine	30	B	AMP FOX ENR CN
21	Swine	30	B	AMP TE CN
22	Swine	35	C	AMP C TE

Legend: AK = amikacin; AMP = ampicillin; AMC = amoxicillin-clavulanate; ATM = aztreonam; CTX = cefotaxime; C = chloramphenicol; EFT = ceftiofur; ENR = enrofloxacin; FOX = cefoxitin; IMP = Imipenem; SXT = trimethoprim-sulfamethoxazole; TE = tetracycline.

**Table 4:** Positive farms, traps, and samples for *Salmonella* spp. and detected serovars.

Farm	Farmed Animals	Traps ID	Samples Type	*Salmonella* Serotype	Antimicrobial Resistance Profile
8	Swine	10	B	London	/
9	Swine	12	A	Rubislaw	AMP TE ENR CN SXT
		12	A	Napoli	/
		12	B	Rubislaw	AMP IMP TE ENR CIP CN SXT
10	Swine	13	A	Kisarawe	/
		13	B	Rubislaw	AK
		13	C	Rubislaw	/
11	Laying hens	14	C	Kentucky	EFT
19	Swine	26	B	Napoli	/
20	Swine	28	A	Napoli	AMP TE
		28	B	R phase	AMP TE
		28	C	Kentucky	/
		28	C	R phase	/
21	Swine	31	B	R phase	AMP EFT TE
		32	A	Rubislaw	/
22	Swine	34	A	London	/

Legend. ID: identification number; AK = amikacin; AMP = ampicillin; CIP = ciprofloxacin; EFT = ceftiofur; CN = gentamicin; ENR = enrofloxacin; IMP = Imipenem; SXT = trimethoprim-sulfamethoxazole; TE = tetracycline.

**Table 5:** Results of disk diffusion test for *Salmonella* spp.

Antimicrobial	Susceptible		Intermediate		Resistant	
	N° of Isolates	%	N° of Isolates	%	N°. of Isolates	%
Ampicillin	11	68.75	0	0.00	5	31.25
Amoxicillin-clavulanate	15	93.75	1	6.25	0	0.00
Cefoxitin	16	100.00	0	0.00	0	0.00
Cefotaxime	12	75.00	4	25.00	0	0.00
Ceftiofur	7	43.75	7	43.75	2	12.50
Imipenem	12	75.00	3	18.75	1	6.25
Ertapenem	16	100.00	0	0.00	0	0.00
Aztreonam	16	100.00	0	0.00	0	0.00
Chloramphenicol	16	100.00	0	0.00	0	0.00
Tetracycline	11	68.75	0	0.00	5	31.25
Enrofloxacin	13	81.25	1	6.25	2	12.50
Ciprofloxacin	14	87.50	1	6.25	1	6.25
Gentamicin	14	87.50	0	0.00	2	12.50
Amikacin	12	75.00	3	18.75	1	6.25
Trimethoprim-sulfamethoxazole	14	87.50	0	0.00	2	12.50

## Data Availability

The data presented in this study are available within the article.
